# Assessment of Neutralizing Antibody Response Against SARS-CoV-2 Variants After 2 to 3 Doses of the BNT162b2 mRNA COVID-19 Vaccine

**DOI:** 10.1001/jamanetworkopen.2022.10780

**Published:** 2022-05-09

**Authors:** Koichi Furukawa, Lidya Handayani Tjan, Yukiya Kurahashi, Silvia Sutandhio, Mitsuhiro Nishimura, Jun Arii, Yasuko Mori

**Affiliations:** 1 Division of Clinical Virology, Center for Infectious Diseases, Kobe University Graduate School of Medicine, Kobe, Japan

## Abstract

**Importance:**

Although 2 and 3 doses of vaccine have been implemented against the SARS-CoV-2 pandemic, the level of immunity achieved by these additional vaccinations remains unclear.

**Objective:**

To investigate the induction of neutralizing antibodies against the SARS-CoV-2 Omicron variant after 2 and 3 doses of the BNT162b2 messenger RNA (mRNA) vaccine among recipients of different ages.

**Design, Setting, and Participants:**

A cohort study was conducted from June 1, 2021, to January 12, 2022, among 82 physicians at Kobe University Hospital who had received 2 doses of the BNT162b2 mRNA vaccine.

**Main Outcomes and Measures:**

The rates of positive test results and the titers of neutralizing antibodies against the Omicron variant after 2 and 3 doses of the vaccine were compared with those against other variants and compared among 3 age groups (≤38 years [younger age group], 39-58 years [intermediate age group], and ≥59 years [older age group]).

**Results:**

A total of 82 physicians (71 men [87%]; median age, 44 years [IQR, 33-58 years]) participated; 31 (38%) were in the younger age group, 32 (39%) were in the intermediate age group, and 19 (23%) were in the older age group. At 2 months after 2 doses of the vaccine, 23 participants (28%) had neutralizing antibodies against the Omicron variant, with a titer of 1.3 (95% CI, 1.2-1.4), which was 11.8-fold (95% CI, 9.9-13.9) lower than the titer against the D614G variant and the lowest among the variants tested. Although the titer of the neutralizing antibody against the Delta variant tended to be low among the older age group (2.9 [95% CI, 2.0-4.1]), the titers of the neutralizing antibody against the Omicron variant were low among all age groups (younger age group, 1.3 [95% CI, 1.1-1.6]; intermediate age group, 1.3 (95% CI, [95% CI, 1.1-1.5]; and older age group, 1.2 [95% CI, 1.0-1.4]). At 7 months after 2 doses of the vaccine, 5 participants (6%) had the neutralizing antibody against the Omicron variant, but after the booster (third dose) vaccination, all 72 participants who received the booster had the neutralizing antibody, and the titer was 41 (95% CI, 34-49), much higher than that at 7 months after 2 doses of the vaccine (1.0 [95% CI, 1.0-1.1]). This increase in titers was observed regardless of age groups; the titers were 44 (95% CI, 32-59) among the younger age group, 44 (95% CI, 32-59) among the intermediate age group, and 30 (95% CI, 22-41) among the older age group.

**Conclusions and Relevance:**

In this cohort study of 82 Japanese participants, 2 doses of the BNT162b2 mRNA vaccine did not induce sufficient neutralizing antibody against the Omicron variant. However, booster vaccination was associated with induction of a high level of neutralizing antibodies against the Omicron variant, irrespective of the recipient’s age.

## Key Points

**Question:**

Are neutralizing antibodies against the Omicron variant of SARS-CoV-2 sufficiently induced after 2 to 3 doses of the BNT162b2 messenger RNA vaccine in recipients of different ages?

**Findings:**

In this cohort study of 82 Japanese participants, 28% and 6% had neutralizing antibodies against the Omicron variant at 2 and 7 months, respectively, after 2 doses of the vaccine; both titer values were low in all age groups. After receiving a booster vaccination, all participants acquired much higher levels of neutralizing antibodies irrespective of age.

**Meaning:**

This study suggests that booster vaccination was associated with induction of higher levels of neutralizing antibodies against the Omicron variant, irrespective of the recipient’s age.

## Teaser

This cohort study investigates the induction of neutralizing antibodies against the SARS-CoV-2 Omicron variant after 2 and 3 doses of the BNT162b2 mRNA vaccine among recipients of different ages.

## Introduction

The COVID-19 pandemic declared by the World Health Organization in March 2020 continues to affect all of the countries around the world. Currently, the Omicron variant (B.1.1.529) of SARS-CoV-2 is rapidly spreading and has become the dominant variant worldwide, including in Japan.^1,2,3,4,5^ In efforts to control the pandemic, several vaccine platforms have been developed using SARS-CoV-2 (Wuhan-1) as the template, and the BNT162b2 messenger RNA (mRNA) vaccine (BioNTech-Pfizer) was granted an emergency use authorization by the US Food and Drug Administration in December 2020, after showing a high level of effectiveness in preventing infection.^6^

However, since April 2020, a variant bearing a D614G variant of the spike protein and several SARS-CoV-2 variants classified as variants of concern or variants of interest by the World Health Organization have emerged and spurred the spread of infection worldwide. As of this writing, SARS-CoV-2 variants classified as variants of concern include Alpha (B.1.1.7), first detected in the UK^7,8,9^; Beta (B.1.351), from South Africa^10,11,12,13^; Gamma (P.1), from Brazil^14,15^; Delta (B.1.617.2), from India^16^; and Omicron (B.1.1.529), from South Africa and Bostwana.^17,18^ The emergence of some variants poses a tremendous challenge because they may reduce the effectiveness of the vaccines.^19^

In particular, the Beta and Gamma variants carry the E484K mutation in the receptor binding domain of the spike protein, which allows the evasion of neutralizing antibodies against the original SARS-CoV-2, further compromising monoclonal antibody therapy.^20,21,22,23^ The Delta variant has L452R, T478K, and P681R mutations in the receptor binding domain, which increases the transmissibility of the virus, leading to a higher viral load in infected individuals.^24,25^ However, the novel Omicron variant is the most mutated variant, with more than 30 mutations in the spike protein and 15 mutations in the receptor binding domain.^26^ There is thus concern about the Omicron variant’s higher transmissibility and increasing immune escapes from COVID-19 vaccines and antibody-based therapies.^27,28,29,30^ It has also been demonstrated that vaccine-induced neutralizing antibody responses decrease over time in individuals who have been vaccinated.^31,32,33^

To counteract the decrease in the neutralizing antibody response over time and the emergence of new variants, including Omicron, a third dose of vaccine, which has been called a *booster vaccination*, has been approved and administered to individuals who were vaccinated more than 6 months prior; the booster vaccination has been shown to effectively induce a high titer of neutralizing antibody.^34^ Recently, some reports showed that the primary vaccine series induced much lower antibody titers or no neutralizing antibodies against the Omicron variant, but individuals who received a booster dose of an mRNA vaccine exhibited potent neutralization of the Omicron variant.^30,35^ However, it is unclear whether the neutralizing antibody titers against the Omicron variant obtained by 2 or 3 doses of the vaccine correlated with the ages of recipients or the appearance of adverse effects.

We investigated the breadth of neutralizing antibodies against the D614G, Alpha, Beta, Gamma, Delta, Kappa, and Omicron variants in serum samples obtained from 82 vaccinated physicians without a history of SARS-CoV-2 infection. In addition, we analyzed the correlations between the neutralizing antibody titers and the ages or adverse effects of the recipients. We also followed up and analyzed the neutralizing antibody titers against the D614G, Delta, and Omicron variants in individuals after they received the booster vaccine.

## Methods

### Participant Recruitment

From June 1 to July 6, 2021, blood samples from 82 physicians at Kobe University Hospital (Kobe, Japan) who had already received 2 doses of the BNT162b2 mRNA COVID-19 vaccine and had never tested positive for SARS-CoV-2 infection were obtained and analyzed (first sampling point). From October 20 to November 1, 2021, blood samples were again obtained from the participants (second sampling point). In addition, blood samples from the 72 participants who received a booster vaccine were obtained in January 2022 and analyzed (third sampling point). This study was approved by the ethical committee of the Kobe University Graduate School of Medicine. All participants were included after providing written informed consent. No statistical methods were used to predetermine the sample size. This study followed the Strengthening the Reporting of Observational Studies in Epidemiology (STROBE) reporting guideline for cohort studies.

### Questionnaire on Adverse Effects

When blood samples were obtained, a questionnaire was used to also obtain data on the participants’ age, sex, and adverse effects that occurred within the first week after each vaccination. Items on the following adverse effects were included: fever (temperature >37.5 °C), general fatigue, and injection site pain (0, none; 1, mild; 2, moderate; and 3, severe). The degree of injection site pain was subjectively determined by each participant.

### Measurement of Neutralizing Activity Against SARS-CoV-2

The neutralization assay was performed using authentic viruses, as recently described.^36,37,38^ In brief, a 2-fold serial dilution of heat-inactivated serum was prepared and mixed with the virus and incubated at 37 °C for 1 hour. This mixture was then added to Vero E6 (transmembrane serine protease 2) cells and incubated for 6 days. The neutralizing antibody titer was determined and shown as the highest serum dilution that did not show any cytopathic effects. The cutoff titer was set at 2 as the detection limit. We previously confirmed this assay by using the serum samples of healthy individuals (n = 24) as a negative control, and we observed that none had neutralizing activity.

### Preparation of SARS-CoV-2 Variants

We used the SARS-CoV-2 Biken-2 strain with the D614G variant as a conventional variant (accession number LC644163); it was provided by the BIKEN Innovative Vaccine Research Alliance Laboratories. The other variants—Alpha (GISAID ID: EPI_ISL_804007), Beta (GISAID ID: EPI_ISL_1123289), Gamma (GISAID ID: EPI_ISL_833366), Delta (GISAID ID: EPI_ISL_2158617), Kappa (GISAID ID: EPI_ISL_2158613), and Omicron (GISAID ID: EPI_ISL_7418017)—had been isolated and were provided by the National Institute of Infectious Diseases, Tokyo, Japan. Each variant was confirmed by the complementary DNA sequence of its spike gene.

### Statistical Analysis

Prism software, version 8.4.3 (GraphPad) and Stata software, version 14.2 (Stata Corp LLC) were used for the statistical analysis and for the preparation of the figures. The geometric mean titer (GMT) of the neutralizing antibody was shown as the representative value with a 95% CI. The neutralizing antibody titers less than 2 were assigned a titer of 1 for the GMT calculations. The Friedman test was used to compare the neutralizing antibody titers among paired groups of participants. The Mann-Whitney test or the Kruskal-Wallis test was used to compare the neutralizing antibody titers or the ages of the different groups of participants. If a significant difference was found by the Friedman test or the Kruskal-Wallis test, the Dunn multiple comparison test was then performed. Results were considered statistically significant when *P* < .05 (2-tailed).

## Results

### Participant Characteristics and Adverse Effects After 1, 2, and 3 Doses of Vaccine

A total of 82 physicians (71 men [87%]; median age, 44 years [IQR, 33-58 years]) participated. Thirty-one (38%) were in the younger age group (≤38 years), 32 (39%) were in the intermediate age group (39-58 years), and 19 (23%) were in the older age group (≥59 years) (Table). The median number of days between the 2 doses of vaccine and between the first and second sampling points was 53 days (IQR, 46-59 days) and 199 days (IQR, 192-203 days), respectively (hereinafter referred to as 2 months and 7 months, respectively, after 2 doses of the vaccine). At the third sampling point, the 72 participants who had received a booster dose of the vaccine were included; the median number of days between the booster vaccine and the third sampling point was 16 days (IQR, 14-21 days).

**Table.** Characteristics of Participants and Adverse Effects of Each Dose of Vaccine

Characteristics	Participants, No. (%)		
	First sampling (n = 82)^a^	Second sampling (n = 82)^b^	Third sampling (n = 72)^c^
Sex			
Male	71 (87)	71 (87)	61 (85)
Female	11 (13)	11 (13)	11 (15)
Age, median (IQR), y	44 (33-58)	44 (33-58)	44 (33-53)
Age group, y			
≤38	31 (38)	31 (38)	31 (43)
39-58	32 (39)	32 (39)	27 (38)
≥59	19 (23)	19 (23)	14 (19)
Time since 2 doses of vaccination, median (IQR), d	53 (46-59)	199 (192-203)	274 (269-278)
Time since booster vaccination, median (IQR), d	NA	NA	16 (14-21)
Adverse effects			
Fever	0	16 (20)	24 (33)
Fatigue	4 (5)	14 (17)	45 (63)
Injection site pain	73 (89)	71 (87)	65 (90)
Mild	30 (37)	27 (33)	15 (21)
Moderate	39 (48)	38 (46)	35 (49)
Severe	4 (5)	6 (7)	15 (21)

Abbreviation: NA, not applicable.

^a^ Samples were collected from June to July 2021; data on adverse effects were obtained after 1 dose of the vaccine.

^b^ Samples were collected from October to November 2021; data on adverse effects were obtained after 2 doses of the vaccine.

^c^ Samples were collected in January 2022; data on adverse effects were obtained after the booster vaccination.

The adverse effects reported by the participants after each vaccination are also shown in the Table. Adverse effects after the booster vaccination were as follows: fever for 24 participants (33%), general fatigue for 45 participants (63%), and injection site pain for 65 participants (90%) (15 mild, 35 moderate, and 15 severe). The frequency of systemic reactions, including fever and fatigue, increased with each successive vaccination, with the greatest frequency being observed after the booster. The frequency of injection site pain did not change. No participant had serious adverse events.

### Neutralizing Activity Against SARS-CoV-2 Variants Compared With D614G at 2 Months After 2 Doses of the Vaccine

**Figure 1.**
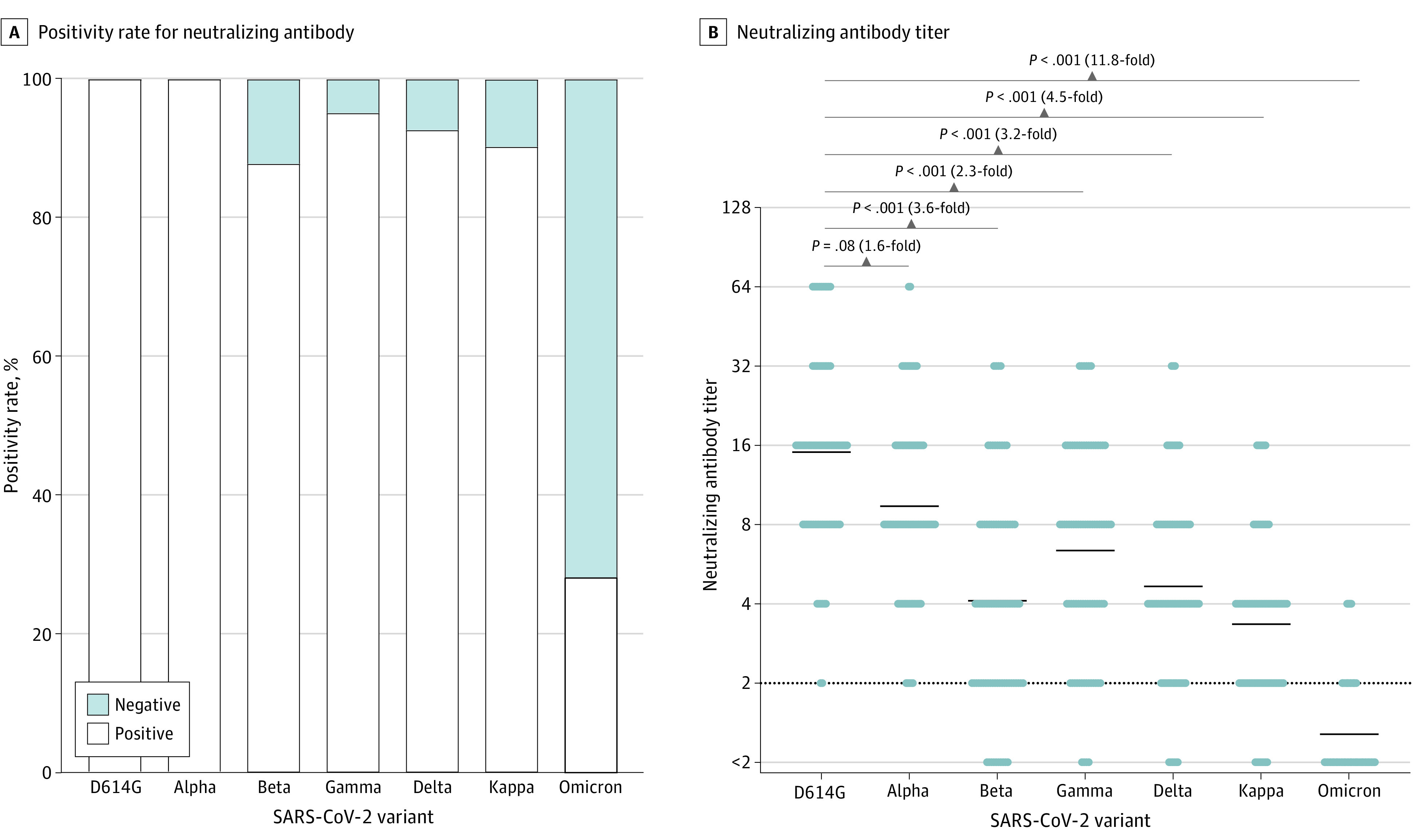
Neutralizing Antibody Against SARS-CoV-2 Variants at 2 Months After 2 Doses of the BNT162b2 mRNA Vaccine The serum samples obtained from 82 recipients were tested for neutralizing activity against the SARS-CoV-2 variants D614G, Alpha, Beta, Gamma, Delta, Kappa, and Omicron. The neutralizing antibody titer is represented by the highest serum dilution that did not show any cytopathic effects. A, The positivity rate for the neutralizing antibody against each variant. Serum samples with neutralizing activity at 2-fold dilution (the detection limit) or higher were considered positive. B, Scatterplot of the neutralizing antibody titers. The limit of detection is indicated by the horizontal dotted black line, and the short horizontal solid black lines indicate the geometric mean titer. The titers against the 7 variants were compared using the Friedman test and the Dunn test for multiple comparisons; 2-tailed *P* values were calculated.

Although the neutralizing antibody titer varied, the serum samples obtained from all 82 participants showed neutralizing activity against the D614G and Alpha variants. In contrast, most serum samples, but not all, showed neutralizing activity against the Beta, Gamma, Delta, and Kappa variants; such activity was observed in 72 participants (88%), 78 participants (95%), 76 participants (93%), and 74 participants (90%), respectively. However, only 23 participants (28%) had neutralizing activity against the Omicron variant (Figure 1A). The GMTs of neutralizing antibody were 15.1 (95% CI, 12.5-18.1) against the D614G variant, 9.4 (95% CI, 7.9-11.2) against the Alpha variant, 4.1 (95% CI, 3.3-5.0) against the Beta variant, 6.4 (95% CI, 5.2-7.8) against the Gamma variant, 4.7 (95% CI, 3.9-5.6) against the Delta variant, 3.3 (95% CI, 2.9-3.9) against the Kappa variant, and 1.3 (95% CI, 1.2-1.4) against the Omicron variant (Figure 1B). The fold reductions of the titers against these variants compared with that against the D614G variant are also shown in Figure 1B; the neutralizing antibody titer against the Omicron variant was 11.8-fold (95% CI, 9.9-13.9) lower than that against the D614G variant and the lowest among all variants tested.

### Neutralizing Activity Against Omicron and the Other Variants at 2 Months After 2 Doses of the Vaccine in Each Age Group

**Figure 2.**
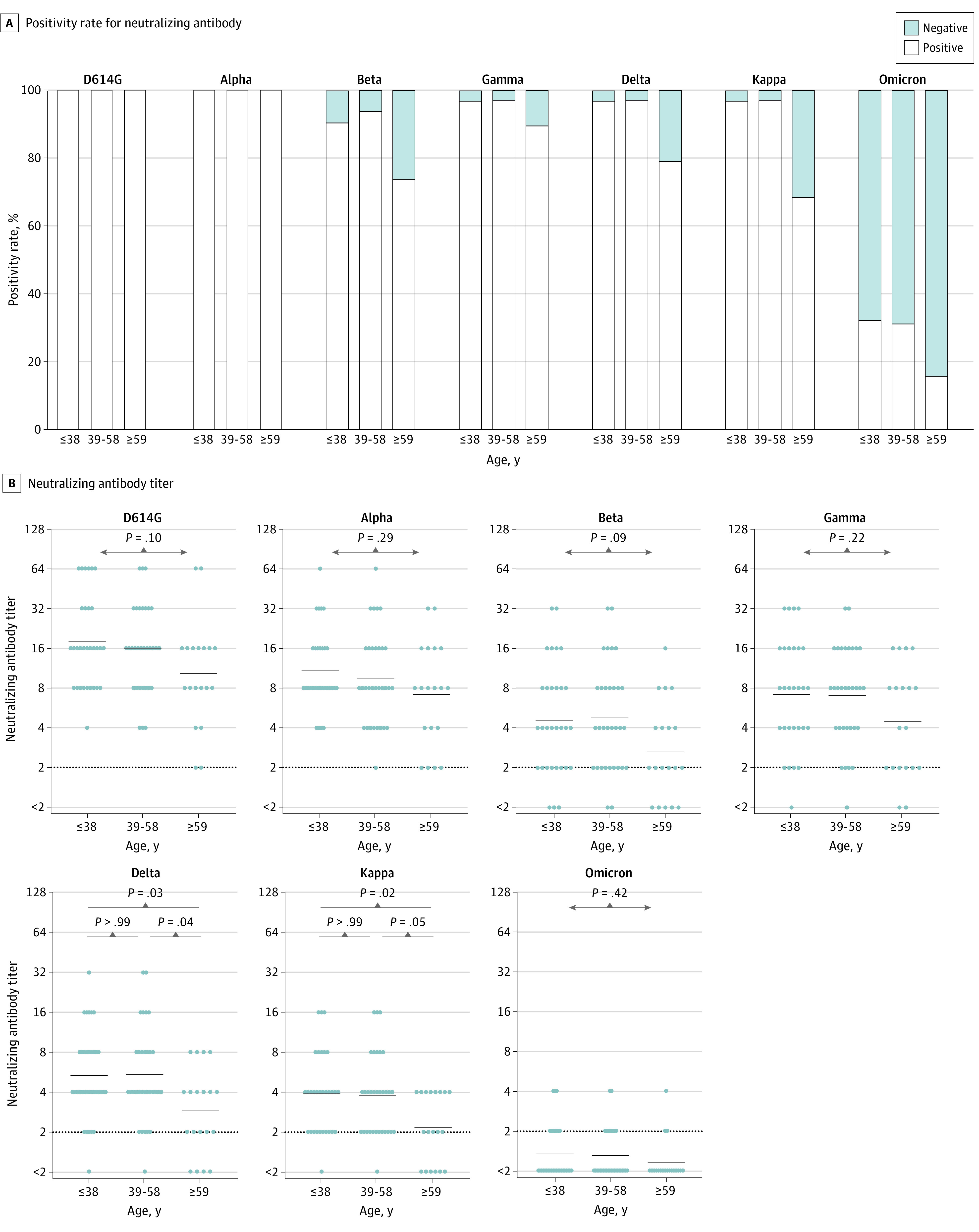
Comparison of Neutralizing Antibodies Against SARS-CoV-2 Variants at 2 Months After 2 Doses of the BNT162b2 mRNA Vaccine Among 3 Age Groups The 82 participants were divided into 3 age groups: 31 participants who were 38 years or younger, 32 participants who were 39 to 58 years, and 19 participants who were 59 years or older. The recipients’ serum samples were then tested for neutralizing activities against the 7 SARS-CoV-2 variants. A, The positivity rate for neutralizing antibodies against each variant in the 3 age groups. Serum samples with neutralizing activity at 2-fold dilution (detection limit) or higher were considered positive. B, Comparison of neutralizing antibody titers against each variant among the age groups. The limit of detection is indicated by the horizontal dotted black line, and the short horizontal solid black lines indicate the geometric mean titer. The Kruskal-Wallis test and the Dunn test for multiple comparisons were performed, and 2-tailed *P* values were calculated.

Although all of the participants’ serum samples showed neutralizing activity against the D614G and Alpha variants irrespective of age, serum samples with negative neutralizing activity against other variants were observed from all age groups, and the proportion of serum samples exhibiting such activity tended to be higher among the older age group (Figure 2A). However, the proportion of serum samples positive for neutralizing antibody against the Omicron variant was low in all 3 age groups; even in the younger age group, only 10 of 31 participants (32%) exhibited positivity for the neutralizing antibody against the Omicron variant. The comparison of neutralizing antibody titers among the 3 age groups yielded results similar to those for the positivity rate; the GMT of the neutralizing antibody against the Delta variant was 2.9 (95% CI, 2.0-4.1) in the older age group, which was significantly lower than those in the 2 other groups: 5.3 (95% CI, 4.0-7.1; *P* = .03) in the younger age group and 5.4 (95% CI, 4.0-7.3; *P* = .04) in the intermediate age group. However, the GMT of the neutralizing antibody against the Omicron variant was substantially lower in all 3 age groups: 1.3 (95% CI, 1.1-1.6) in the younger age group, 1.3 (95% CI, 1.1-1.5) in the intermediate age group, and 1.2 (95% CI, 1.0-1.4) in the older age group (Figure 2B).

### Neutralizing Activity Against 3 Variants at 7 Months After 2 and 3 Doses of the Vaccine

**Figure 3.**
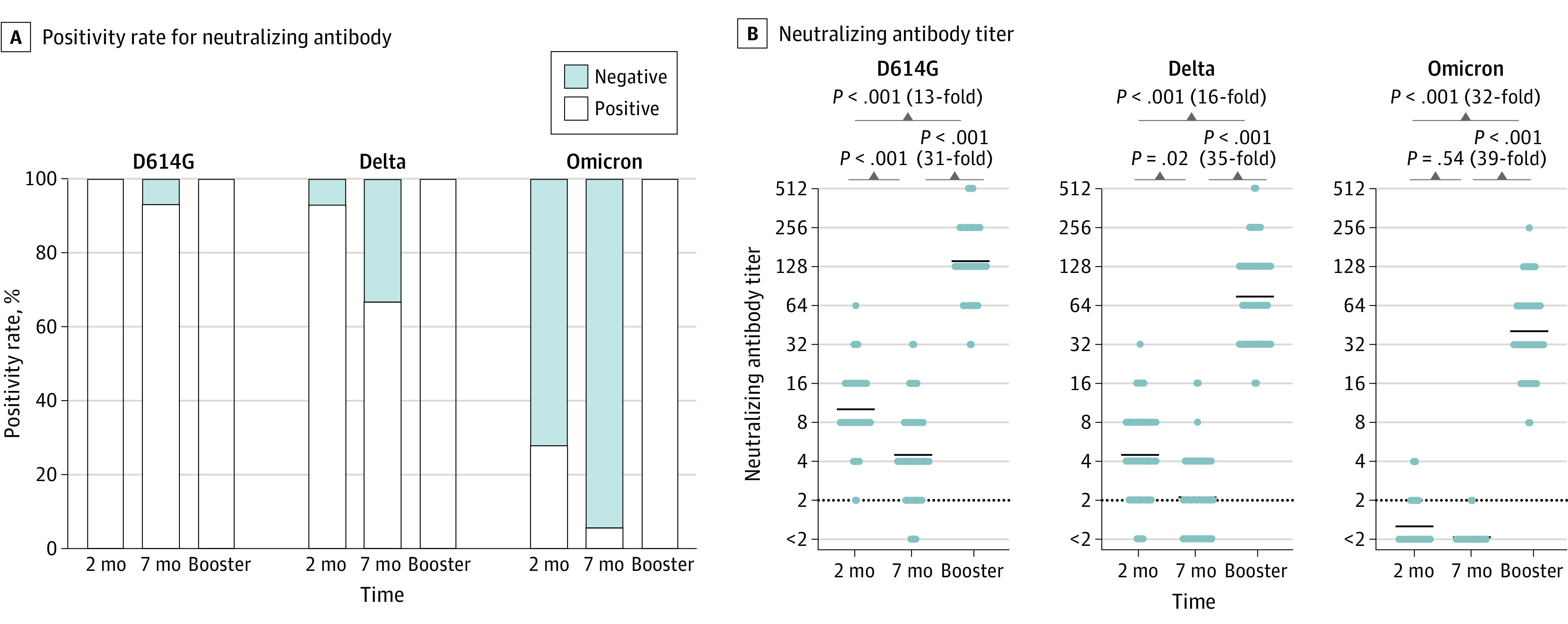
Changes in the Neutralizing Antibody Against 3 SARS-CoV-2 Variants After Booster Vaccination The serum samples obtained from the 72 participants who received the booster vaccination were tested for neutralizing activity against the D614G, Delta, and Omicron variants at 2 and 7 months after 2 doses of the BNT162b2 mRNA vaccine and after the booster (3 total doses of vaccine). A, Changes in the positivity rates for neutralizing antibodies against each variant. Serum samples with neutralizing activity at 2-fold dilution (detection limit) or higher were considered positive. B, Changes in the neutralizing antibody titers against each variant. The limit of detection is indicated by the horizontal dotted black line, and the short horizontal solid black lines indicate the geometric mean titer. The titers were compared using the Friedman test and the Dunn test for multiple comparisons; 2-tailed *P* values were calculated.

At 7 months after 2 doses of the vaccine, the serum samples obtained from 76 (93%), 55 (67%), and 5 (6%) of the 82 participants had neutralizing activity against the D614G, Delta, and Omicron variants, respectively (Figure 3A). After the booster vaccination, however, the serum samples obtained from all 72 participants who had received the booster vaccination had neutralizing activity against these 3 variants. Figure 3B illustrates the changes in the neutralizing antibody titers against these variants during the same period. The GMTs of the neutralizing antibody against the D614G and Delta variants were significantly decreased at 7 months (D614G variant, 4.5 [95% CI, 3.7-5.4]; Delta variant, 2.1 [95% CI, 1.8-2.5]) compared with those at 2 months after 2 doses of the vaccine. Although the titer against the Omicron variant also tended to decrease during this period (1.0 [95% CI, 1.0-1.1]), no significant reduction was observed. The GMTs of the neutralizing antibody after the booster vaccination were much higher than those at 2 and 7 months after 2 doses of the vaccine against all variants (ie, 140 [95% CI, 121-164] against the D614G variant, 75 [95% CI, 62-91] against the Delta variant, and 41 [95% CI, 34-49] against the Omicron variant) (Figure 3B).

### Comparison of Neutralizing Antibody Titers After the Booster Vaccination in the 3 Age Groups

**Figure 4.**
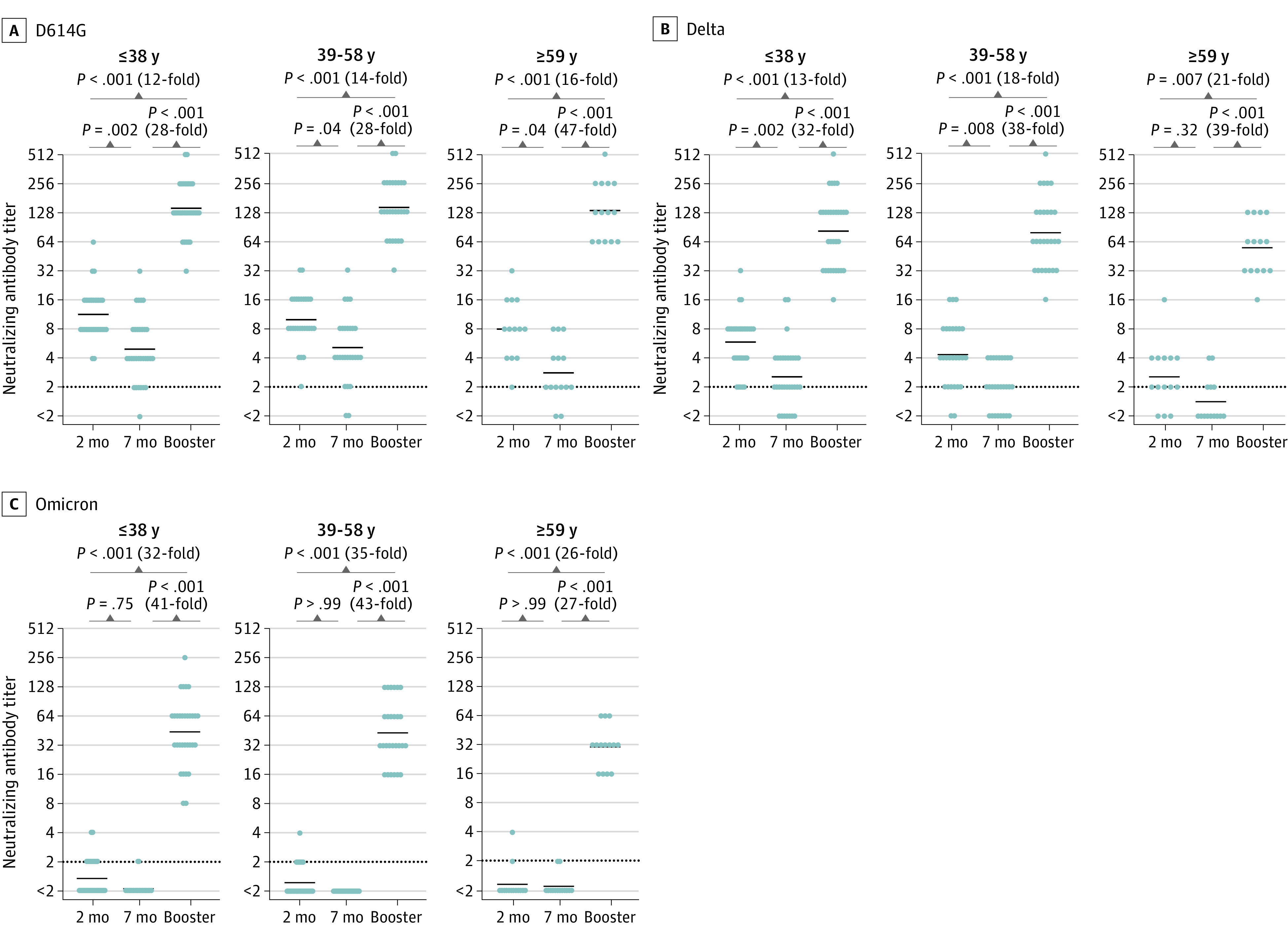
Comparison of Changes in the Neutralizing Antibody Titer After Booster Vaccination Among the 3 Age Groups The 72 recipients who received the booster vaccination were divided into 3 age groups: 31 participants who were 38 years or younger, 27 participants who were 39 to 58 years, and 14 participants who were 59 years or older. The neutralizing antibody titers against the D614G variant (A), the Delta variant (B), and the Omicron variant (C) at 2 and 7 months after 2 doses of the BNT162b2 mRNA vaccine and after the booster vaccination were compared in each age group. The limit of detection is indicated by the horizontal dotted black line, and the short horizontal solid black lines indicate the geometric mean titer. The Friedman test and the Dunn test for multiple comparisons were performed; 2-tailed *P* values were calculated.

We compared the neutralizing antibody titers after the booster vaccination in each age group (Figure 4A-C). At 7 months after 2 doses of the vaccine, the GMTs of the neutralizing antibody against the D614G and Delta variants tended to be low in the older age group (D614G variant, 2.8 [95% CI, 1.9-4.3]; Delta variant, 1.4 [95% CI, 1.0-1.9]), and those against the Omicron variant were much lower in all 3 age groups. However, after the booster vaccination, the titers greatly increased for all 3 variants, including Omicron, regardless of the ages of participants. The GMTs of the neutralizing antibody after the booster vaccination in the younger, intermediate, and older age groups were 143 (95% CI, 114-180), 141 (95% CI, 108-186), and 134 (95% CI, 90-200), respectively, against the D614G variant; 82 (95% CI, 60-112), 79 (95% CI, 56-110), and 55 (95% CI, 31-81), respectively, against the Delta variant; and 44 (95% CI, 32-59), 44 (95% CI, 32-59), and 30 (95% CI, 23-41), respectively, against the Omicron variant.

### Adverse Effect–Related Differences in Neutralizing Activity and Recipient Age

In the comparison of neutralizing antibody titers at 2 months after 2 doses of the vaccine against the D614G variant, there was no significant difference between the participants with and the participants without adverse effects, including fever, injection site pain, and fatigue (eFigure 1A in the Supplement). However, the comparison of participants’ ages and adverse effects revealed that the median age of the participants with fever was significantly younger than that of the participants without fever (37 years [range, 28-64 years] vs 46 years [range, 29-68 years]; *P* = .02). There were no significant differences in the median age between the participants with and the participants without fatigue (44 years [range, 28-68 years] vs 39 [range, 28-64 years]; *P* = .97) or regarding the degrees of injection site pain (none: 41 years [range, 31-68 years]; mild: 50 years [range, 30-64 years]; moderate: 42 years [range, 28-64 years]; severe: 39 years [range, 36-51] years; *P* = .29) (eFigure 1B in the Supplement). In the same comparison after the booster vaccination, however, there was no significant correlation between adverse effects and neutralizing antibody titers or the age of recipients (eFigure 2A and 2B in the Supplement).

## Discussion

We evaluated neutralizing activities against authentic SARS-CoV-2 variants in the serum samples obtained from adults vaccinated with 2 or 3 doses of the BNT162b2 mRNA vaccine. We also analyzed the changes in the neutralizing antibody titers after the booster vaccination in different age groups, as well as the associations between adverse effects, the age of participants, and the neutralizing antibody titers.

Our results demonstrated that only 28% of the participants had neutralizing antibodies against the Omicron variant at 2 months after 2 doses of the vaccine, and the titer against the Omicron variant was 11.8-fold lower than that against the D614G variant and the lowest among the variants tested, suggesting that the neutralizing antibody against the Omicron variant cannot be sufficiently induced by 2 doses of the vaccine. Recently, other groups have reported similar results of a large (eg, 22-fold^30^ or 23-fold^39^) reduction in the neutralization of the Omicron variant compared with the ancestral virus induced by vaccine or previous infection, and our results reflected these reports despite our use of a different neutralization method based on 100% inhibition criteria.^28,29,30,39,40,41^ At 7 months after 2 doses of the vaccine, that tendency was even more pronounced, with only 6% of the participants showing neutralizing antibodies against the Omicron variant, suggesting a high risk of breakthrough infection or reinfection. This finding was consistent with a recent report in which serum samples showed that the vaccine barely inhibited the Omicron variant at 5 months after 2 doses.^42^

Regarding the potential cause of this finding, it has been reported that mutations on the spike protein of the Omicron variant, especially the K417N, N440K, G446S, S477N, T478K, E484A, Q493R, G496S, Q498R, N501Y, and Y505H mutations, were responsible for the Omicron variant’s broad evasion from antibodies.^43^ In addition, the Omicron variant also has multiple mutations at the proximal to N343 glycan and N-terminal domain, which could be the target of potent neutralizing antibodies.^29,35,44^ In the case of the Delta variant, the L452R, T478K, and P681R mutations have been reported to promote the interaction between the spike and angiotensin-converting enzyme 2 receptor by inducing structural changes in the binding domain, resulting in immune evasion and increased transmissibility.^45,46,47^ In our study, 67% of the participants had neutralizing antibodies against the Delta variant at 7 months after 2 doses of the vaccine, suggesting that neutralizing antibodies against the Delta variant could be more easily induced and retained by 2 doses of the vaccine compared with the Omicron variant.

We also observed much higher levels of neutralizing antibodies against the Omicron variant after the booster vaccination compared with those after 2 doses of the vaccine. This finding may indicate that the booster vaccination achieves a sufficient induction of immunity against the Omicron variant and would likely achieve sufficient immunity against other future variants by inducing neutralizing antibodies that recognize common epitopes and by promoting affinity maturation of the antibody.^48,49,50,51^

Our comparison of neutralizing antibody titers among the 3 age groups at 2 months after 2 doses of the vaccine showed that neutralizing activity against all 7 tested variants tended to be lower in older participants (with significantly lower activity being observed only for the Delta and Kappa variants). This result was consistent with previous reports showing that people older than 60, 65, or 80 years of age vaccinated with a 2-shot series produced significantly fewer neutralizing antibodies than similarly vaccinated individuals in other age groups.^33,52,53^ However, our finding of no significant difference in the neutralizing antibody titers against the Omicron variant among these age groups reflects the universally low neutralizing activity for the Omicron variant, unlike that for the Delta variant.

In contrast, the increase in the neutralizing antibody titers after the booster vaccination did not show a large difference among the 3 age groups, and the neutralizing antibody titer against the Omicron variant was sufficiently high in all age groups, suggesting that the booster vaccination could be recommended for all individuals regardless of age.

In this study, the frequency of systemic adverse effects, including fever and fatigue, increased with each additional dose of the vaccine, with such effects being most frequent after the booster vaccination. This finding indicates that the systemic adverse effects may increase as a consequence of recall responses for the 2 and 3 doses.^54,55,56,57^ However, we did not detect a correlation between the neutralizing antibody titers and the adverse effects of each vaccination, suggesting the necessity of investigating the association between humoral immunity and immunologic memory.^58^

### Limitations

This study has some limitations. First, most participants were male, which may have somewhat weakened the universality of our results. Second, because information about the medical histories, comorbid conditions, and medications of each participant could not be obtained, it is possible that any of these factors might have affected the results of neutralizing antibody titers.^59^

## Conclusions

Our cohort study of vaccinated participants showed that a sufficient neutralizing antibody against the Omicron variant could not be induced by 2 doses of the BNT162b2 mRNA vaccine, suggesting a high risk of breakthrough infection or reinfection, especially for those 59 years of age or older. Although systemic adverse effects, such as fever and general fatigue, may increase, the booster vaccination was associated with a strong induction of neutralizing antibodies against the Omicron variant regardless of the age of the recipients. The booster vaccination could therefore be recommended to prevent the further spread of COVID-19.

## Notes

Supplement. **eFigure 1.** Comparison of Adverse Effects and Neutralizing Antibody or the Age of Recipients After Two Doses of Vaccine **eFigure 2.** Comparison of Adverse Effects and Neutralizing Antibody or the Age of Recipients After the Booster Vaccine
