# Supplementary material for: Assessment of Neutralizing Antibody Response Against SARS-CoV-2 Variants After 2 to 3 Doses of the BNT162b2 mRNA COVID-19 Vaccine
Source: JAMA Netw Open. 2022 May 9;5(5):e2210780. doi: 10.1001/jamanetworkopen.2022.10780 (PMC9086840; doi:10.1001/jamanetworkopen.2022.10780)
Supplement: Supplement. — eFigure 1. Comparison of Adverse Effects and Neutralizing Antibody or the Age of Recipients After Two Doses of Vaccine eFigure 2. Comparison of Adverse Effects and Neutralizing Antibody or the Age of Recipients After the Booster Vaccine [file jamanetwopen-e2210780-s001.pdf]

Supplementary Online Content

Furukawa K, Tjan LH, Kurahashi Y, et al. Assessment of neutralizing antibody response against SARS-CoV-2 variants after 2 to 3 doses of the BNT162b2 mRNA COVID-19 vaccine. *JAMA Netw Open.* 2022;5(5):e2210780.
doi:10.1001/jamanetworkopen.2022.10780

eFigure 1. Comparison of Adverse Effects and Neutralizing Antibody or the Age of Recipients After Two Doses of Vaccine
eFigure 2. Comparison of Adverse Effects and Neutralizing Antibody or the Age of Recipients After the Booster Vaccine

This supplementary material has been provided by the authors to give readers additional information about their work.

eFigure 1. Comparison of Adverse Effects and Neutralizing Antibody or the Age of Recipients After Two Doses of Vaccine

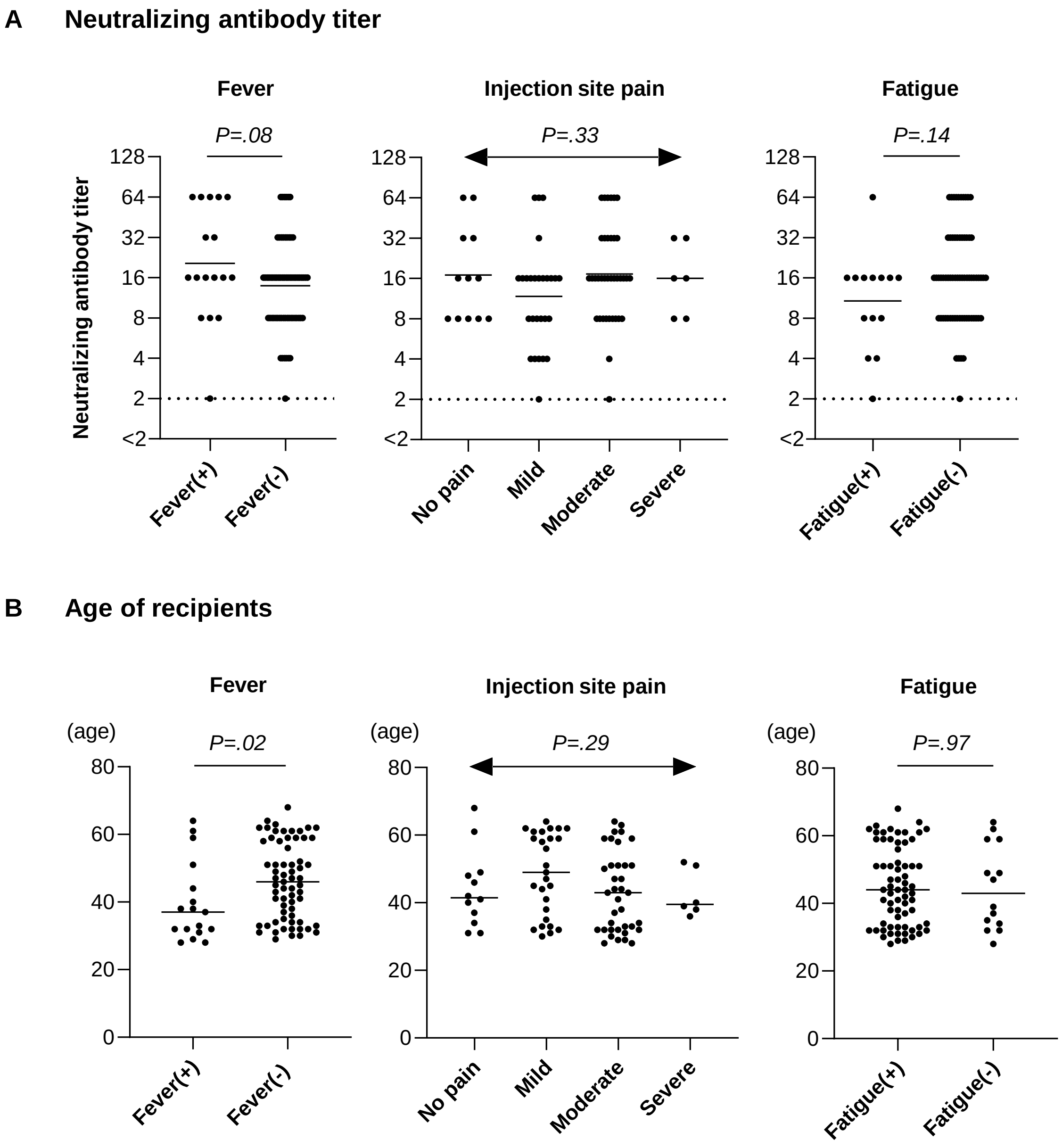

Eighty-two recipients were divided according to the appearance of fever, fatigue, and the degree of injection site pain after two doses of vaccination. **(A)** Comparison between the neutralizing antibody titers and side effects. The limit of antibody detection is displayed with a dotted horizontal line and the solid line shows the geometric mean titers. **(B)** Comparison between the age and side effects of recipients. Horizontal lines indicate the median age of recipients in each group. Mann-Whitney U test was performed for the comparison by the presence or absence of fever and fatigue, and Kruskal-Wallis test was performed for the comparison by the degree of injection site pain. Two-tailed p-values were calculated.

© 2022 Furukawa K et al. *JAMA Network Open.*

eFigure 2. Comparison of Adverse Effects and Neutralizing Antibody or the Age of Recipients After the Booster Vaccine

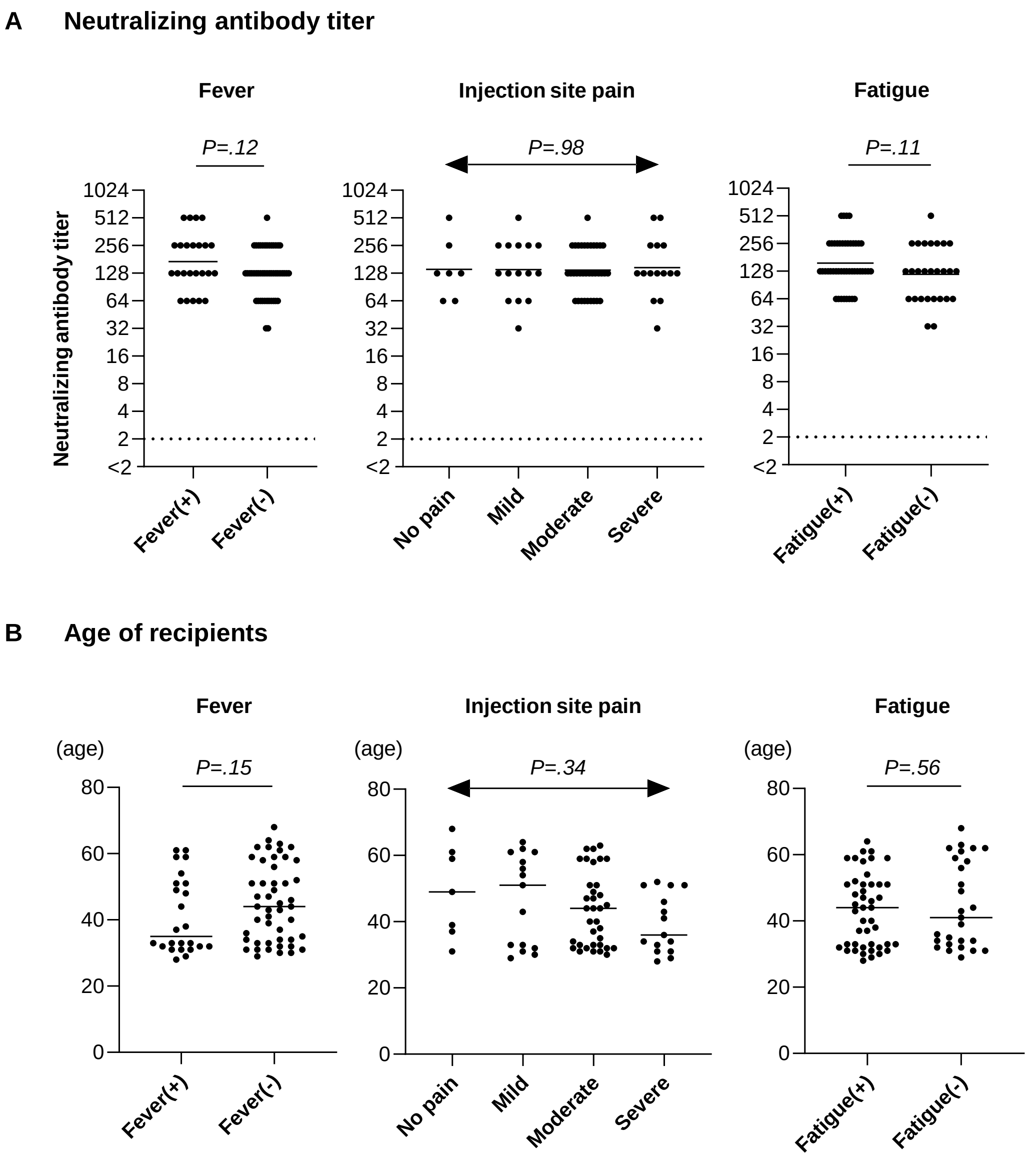

Eighty-two recipients were divided according to the appearance of fever, fatigue, and the degree of injection site pain after the booster vaccination. **(A)** Comparison between the neutralizing antibody titers and side effects. The limit of antibody detection is displayed with a dotted horizontal line and the solid line shows the geometric mean titers. **(B)** Comparison between the age and side effects of recipients. Horizontal lines indicate the median age of recipients in each group. Mann-Whitney U test was performed for the comparison by the presence or absence of fever and fatigue, and Kruskal-Wallis test was performed for the comparison by the degree of injection site pain. Two-tailed p-values were calculated.

© 2022 Furukawa K et al. *JAMA Network Open.*
